# The cysteine proteinase inhibitor Z-Phe-Ala-CHN_2 _alters cell morphology and cell division activity of *Trypanosoma brucei *bloodstream forms *in vivo*

**DOI:** 10.1186/1475-9292-6-2

**Published:** 2007-02-28

**Authors:** Stefan Scory, York-Dieter Stierhof, Conor R Caffrey, Dietmar Steverding

**Affiliations:** 1 Abteilung Parasitologie, Hygiene-Institut der Ruprecht Karls-Universität, Im Neuenheimer Feld 324, 69120 Heidelberg, Germany; 2 Abteilung Membranbiochemie, Max-Planck-Institut für Biologie, Corrensstraße 38, 72076 Tübingen, Germany; 3 Abteilung Tropenhygiene und Öffentliches Gesundheitswesen, Hygiene-Institut der Ruprecht Karls-Universität, Im Neuenheimer Feld 324, 69120 Heidelberg, Germany; 4 Zentrum für Molekularbiologie der Pflanzen, Eberhard-Karls-Universität, Auf der Morgenstelle 1, 72076 Tübingen, Germany; 5 Sandler Center for Basic Research in Parasitic Diseases, California Institute for Quantitative Biomedical Research, Byers Hall, University of California San Francisco, 1700 4th Street, San Francisco, CA94158-2330, USA; 6 Present address: BioMedical Research Centre, School of Medicine, Health Policy and Practice, University of East Anglia, Norwich NR4 7TJ, UK

## Abstract

**Background:**

Current chemotherapy of human African trypanosomiasis or sleeping sickness relies on drugs developed decades ago, some of which show toxic side effects. One promising line of research towards the development of novel anti-trypanosomal drugs are small-molecule inhibitors of *Trypanosoma brucei *cysteine proteinases.

**Results:**

In this study, we demonstrate that treatment of *T. brucei*-infected mice with the inhibitor, carbobenzoxy-phenylalanyl-alanine-diazomethyl ketone (Z-Phe-Ala-CHN_2_), alters parasite morphology and inhibits cell division. Following daily intra-peritoneal administration of 250 mg kg^-1 ^of Z-Phe-Ala-CHN_2 _on days three and four post infection (p.i.), stumpy-like forms with enlarged lysosomes were evident by day five p.i. In addition, trypanosomes exposed to the inhibitor had a 65% greater protein content than those from control mice. Also, in contrast to the normal 16% of parasites containing two kinetoplasts – a hallmark of active mitosis, only 4% of trypanosomes exposed to the inhibitor were actively dividing, indicating cell cycle-arrest.

**Conclusion:**

We suggest that inhibition of endogenous cysteine proteinases by Z-Phe-Ala-CHN_2 _depletes the parasite of essential nutrients necessary for DNA synthesis, which in turn, prevents progression of the cell cycle. This arrest then triggers differentiation of the long-slender into short-stumpy forms.

## Background

*Trypanosoma brucei *is the aetiological agent of human African trypanosomaisis or sleeping sickness. At present there are only four drugs available for treatment of sleeping sickness and some of these induce serious side effects [1]. With this in mind, recent research has shown that small-molecule inhibitors of Clan CA cysteine proteinases [2,3] kill *T. brucei in vitro *and alleviate parasitiemia in mouse models of the disease [4-7]. As possible targets for these inhibitors, two cysteine proteinases have been identified. The first, an ortholog of mammalian cathepsin B (tbcatB), is a single copy gene and expressed in both procyclic and bloodstream forms, but with greater detectable mRNA levels in the latter stage [8]. As yet, its sub-cellular localization is unclear but may be in either the endosome and/or lysosome. Tetracycline-induced RNAi of tbcatB resulted in dysmorphic parasites leading to cell death [8], raising the possibility that tbcatB may be a useful molecular target for disease intervention.

The second potential target for cysteine proteinase inhibitors, termed trypanopain-Tb [5], brucipain [6] or rhodesain [9], is a cathepsin L-like cysteine proteinase [10,11] encoded by 11 gene copies [12] and predominant in terms of enzymatic activity [9]. Inhibition of brucipain by the small molecule inhibitor, carbobenzoxy-phenylalanyl-alanine-diazomethyl ketone (Z-Phe-Ala-CHN_2_), correlated with the compound's trypanocidal action *in vivo *[4]. Also, this and other peptidyl inhibitors blocked proteinolysis in the lysosome as evidenced by the accumulation of undigested FITC-transferrin [4,7], data consistent with the lysosomal localization of brucipain using specific antibodies [9,13]. Brucipain is developmentally expressed, with approximately five-fold more protein found in short-stumpy forms than in either long-slender or procyclic forms [9].

Here, we demonstrate that Z-Phe-Ala-CHN_2 _when administered to mice infected with *T. brucei *results in parasites with altered cell morphology, a decreased capacity to degrade intracellular protein and an inability to mitotically replicate. We discuss these findings with respect to the parasite proteases targeted by Z-Phe-Ala-CHN_2_.

## Results

To study the effect of Z-Phe-Ala-CHN_2 _on the cell morphology and cell division activity of bloodstream-form trypanosomes *in vivo*, mice infected with *T. brucei *were injected i.p. once daily on days 3 and 4 p.i. with 250 mg kg^-1 ^of the inhibitor or vehicle alone. On day 5 p.i., blood smears were prepared and parasites were isolated from infected blood.

**Figure 1:**
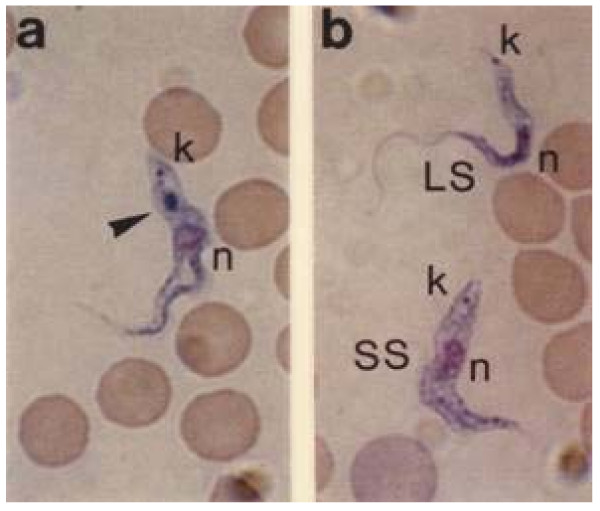
**Effect of Z-Phe-Ala-CHN**_**2 **_**on the morphology of T. *brucei *bloodstream forms *in vivo***. Mice that had been infected with the pleomorphic variant clone AnTat 1.1 were injected intraperitoneally with 250 mg kg^-1 ^of Z-Phe-Ala-CHN_2 _or vehicle alone on days 3 and 4 p.i. On day 5 p.i., blood smears were prepared and stained with May-Grünwald's stain solution. Representative examples from Z-Phe-Ala-CHN_2_-treated mice (**a**) and control mice (**b**) are shown. Trypanosomes exposed to the inhibitor appeared stumpy-like with a blue-stained region (arrowhead) between the kinetoplast and the nucleus, a location that is consistent with that of the lysosome in bloodstream forms. k, kinetoplast; n, nucleus; LS, long-slender forms; SS, short-stumpy forms.

For examining the cell morphology of the parasites by light microscopy, blood smears were stained with May-Grünwald dye. In the blood of control mice, a mixed population of dividing long-slender forms and cell-arrested short-stumpy forms was found (Fig. 1b), with significantly (four times) more long-slender forms. In contrast, the blood of Z-Phe-Ala-CHN_2_-treated mice contained few long-slender forms and almost all trypanosomes (>90%) appeared as stumpy-like forms (Fig. 1a). In addition, a large blue-stained region was observed between the kinetoplast and the nucleus, i.e., in a position consistent with that of the lysosome (Fig. 1a). That this is the lysosome is corroborated by the fact that the May-Grünwald dye stains acidic cell components. Long-slender and short-stumpy forms from control mice did not contain this structure (Fig. 1b).

**Figure 2:**
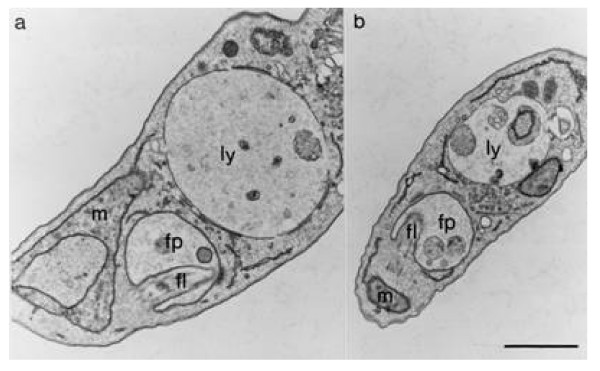
**Effect of Z-Phe-Ala-CHN**_**2 **_**on the size of the lysosome of *T. brucei *bloodstream forms *in vivo***. Mice were infected and treated as described in the legend to Fig. 1. On day 5 p.i., trypanosomes were purified and processed for electron microscopy. Ultrathin sections of representative cells purified from mice treated with Z-Phe-Ala-CHN_2 _(**a**) and vehicle alone (**b**) are shown. Note the enlarged lysosome in the trypanosome exposed to Z-Phe-Ala-CHN_2 _compared with that in the short-stumpy form from control mice. fl, flagellum; fp, flagellar pocket; ly, lysosome, m, mitochondrion. Bar, 0.5 μm.

Upon electron microscopy, trypanosomes from Z-Phe-Ala-CHN_2_-treated mice were considerably larger than those from control mice (Fig. 2). Also, the lysosomes of trypanosomes exposed to the inhibitor were significantly larger than those of short-stumpy forms from control mice (Fig. 2). The enlargement of the lysosome may also explain why this organelle could be easily observed by light microscopy after May-Grünwald staining. In addition, the mitochondrion were also enlarged (Fig. 2).

Next, the protein content of trypanosomes purified from Z-Phe-Ala-CHN_2_-treated and control mice was compared. Trypanosomes exposed to the inhibitor had 65% more protein than parasites from untreated animals; the mean values were 8.9 and 5.4 pg cell^-1^, respectively (Table 1). Thus, the microscopically observed enlargement of trypanosomes exposed to Z-Phe-Ala-CHN_2 _correlated with a higher protein content of the cells.

**Table 1:** Protein content of *T. brucei *bloodstream forms purified from Z-Phe-Ala-CHN_2_-treated and control mice.

	Protein content (pg cell^-1^)	
	
	Z-Phe-Ala-CHN_2_-exposed trypanosomes	Control trypanosomes
Experiment 1	9.1	6.5
Experiment 2	8.7	4.2

Average	8.9	5.4

To determine the number of dividing cells, blood smears were stained with the DNA-binding fluorochrome DAPI and examined by fluorescence microscopy. Trypanosomes were considered to be dividing if the parasites contained two kinetoplasts. In contrast to the 16% of the normal trypanosome population containing two kinetoplasts, just 4% of parasites exposed to Z-Phe-Ala-CHN_2 _were dividing (Table 2). As the segregation of the kinetoplast precedes trypanosomal cytokinesis [14], this result indicates that the cell division of trypanosomes was impaired under the influence of Z-Phe-Ala-CHN_2_.

**Table 2:** Number of *T. brucei *bloodstream forms purified from Z-Phe-Ala-CHN_2_-treated and control mice with two kinetoplasts.

	Two kinetoplast configuration (%) *	
	
	Z-Phe-Ala-CHN_2_-exposed trypanosomes	Control trypanosomes
Experiment 1	3.4	14.9
Experiment 2	4.8	16.4

Average	4.1	15.7

* Analysis of DAPI stained trypanosomes.

## Discussion

Previously, we demonstrated that small molecule inhibitors of cysteine proteinases kill *T. brucei *in culture and experimentally-infected mice [4,6]. We now report that upon treatment of infected mice with the diazomethyl ketone inhibitor, Z-Phe-Ala-CHN_2_, parasite death is preceded by an increase in cell body mass and enlargement of constituent organelles (lysosome and mitochondrion) with a predominance (>90%) of trypanosomes displaying a "stumpy-like" morphology. Swelling of the cell body prior to cell lysis has been reported previously for bloodstream forms of *T. brucei *and *T. cruzi *after incubation with peptidyl fluoromethyl ketones *in vitro *[15]. The mechanism proposed involved inhibition of cysteine proteinase activity.

Treatment with Z-Phe-Ala-CHN_2 _elicited a striking enlargement of the lysosome of trypanosomes coincident with the appearance of the same organelle after staining with May-Grünwald's solution. This suggests that the inhibitor prevents normal proteolysis in the lysosome thereby allowing the accumulation of undegraded proteins and the consequent increase in parasite weight (Table 1). The alteration in lysosomal size and function is consistent with the previous finding that co-incubation of cultured *T. brucei *bloodstream forms with Z-Phe-Ala-CHN_2 _and FITC-labelled transferrin prevented degradation of the latter in the lysosome [4]. However, the lack of increased electron density in the enlarged lysosome, as would normally be expected upon accumulation of undegraded proteins, may suggest an increased water permeability of the organelle.

While it is formally possible that Z-Phe-Ala-CHN_2 _exerts its trypanocidal action through one or more off-target mechanisms, one likely molecular target responsible for the enlarged lysosome phenotype is brucipain given that it is localized in the lysosome [9] and that exposure to the inhibitor *in vivo *results in a marked decrease (92%) in cellular cysteine protease activity, most of which is due to brucipain [4]. It is also possible that the phenotype was a result of inhibition of tbcatb by Z-Phe-Ala-CHN_2_, even though a sub-cellular localization of this enzyme consistent with the phenotype is as yet unknown [8]. Interestingly, tetracycline-induced RNAi of tbcatB, but not brucipain, induced a lethal phenotype prefaced by an enlarged endosome/lysosome compartment [8] similar to that consequent on exposure to Z-Phe-Ala-CHN_2_. The conclusions were that tbcatb, not brucipain, was essential to *T. brucei *survival and that tbcatb was the most likely target of the inhibitor [8]. However, with respect to brucipain, both of these judgments are open to reinterpretation given the available data. First, fully 35% of rhodesain activity remained in the presence of tetracycline-induced RNAi [8], possibly sufficient to allow for normal cell function and the lack of an obvious phenotype. Therefore, it is still unclear what a total knock-down of brucipain might yield in terms of the parasite's ability to survive. Secondly, Z-Phe-Ala-CHN_2 _is chemically reactive with both mammalian cathepsins B and L [16,17] and there is no quantitative data to suggest that tbcatb is preferentially inhibited by this compound. Indeed, it has been shown that, in *T. brucei *lysates, both brucipain and a 34 kDa proteinase species (consistent with the molecular weight of tbcatb) are inhibited by Z-Phe-Ala-CHN_2 _[9].

For other protozoan parasites, morphological aberrations, consistent with the prevention of normal proteinolysis, have been noted upon application of cysteine proteinase inhibitors. Thus, incubation of *T. cruzi *epimastigotes with the cysteine proteinase inhibitor morpholinourea-phenylalany-homophenylalanine vinylsulfone phenyl (K11777) led to enlarged intracellular organelles (endoplasmatic reticulum, nuclear membrane, mitochondrion) and morphological alterations of the Golgi complex [18]. Likewise, for *Plasmodium falciparum *trophozoites, cysteine proteinase inhibitors disrupted the morphology of the food vacuole and prevented degradation of haemoglobin [19,20].

In addition to the morphological changes, the "stumpy-like" nature of trypanosomes exposed to Z-Phe-Ala-CHN_2 _was substantiated by the low number of dividing parasites. Only 4% of the parasites were proliferating which is close to the number of dividing cells (long-slender forms) of about 2% found in natural short-stumpy enriched populations *in vivo *[21]. Because we observed no increase in multinucleated cells with aberrant kinetoplast/nucleus configurations, as can occur under non-physiological conditions [22,23], the low number of dividing cells indicates Z-Phe-Ala-CHN_2 _induces a cell cycle arrest.

Transformation of long-slender forms into "stumpy-like" forms has been previously observed upon treatment with the methylating agent 1,2-bis(methylsulfonyl)-1-methylhydrazine and the ornithine decarboxylase inhibitor DL-α-difluoromethylornithine (DFMO) [22,24,25]. Whereas the primary effect of DFMO is depletion of the intracellular polyamine pool, that of 1,2-bis(methylsulfonyl)-1-methylhydrazine is modification of DNA. However, the subsequent effect of both agents is an inhibition of DNA synthesis which in turn leads to arrest of the cell cycle [22,25]. A similar mechanism may also account for the cell cycle arrest in trypanosomes exposed to Z-Phe-Ala-CHN_2_: inhibition of lysosomal proteolysis depletes the parasite of nutrients necessary for DNA synthesis and this is followed by blockage of mitosis. The cell-cycle arrest may also explain why Z-Phe-Ala-CHN_2_-exposed trypanosomes are 65% larger than control parasites as they continue to grow but have stopped dividing.

## Conclusion

This study has shown that treatment of *T. brucei*-infected mice with the cysteine proteinase inhibitor Z-Phe-Ala-CHN_2 _results in an increased number of mitotically-arrested, stumpy form-like parasites. The findings agree with previous suggestions that enforced cell cycle arrest can trigger slender-to-stumpy differentiation [21].

## Materials and methods

### Reagents

Z-Phe-Ala-CHN_2 _was purchased from Bachem, Heidelberg, Germany; May-Grünwald's stain solution was obtained from Merck, Darmstadt, Germany; 4,6-diamidino-2-phenylinodole (DAPI) was bought from Sigma, Deisenhofen, Germany; BCA Protein Assay was from Pierce Chemical Company (Rockford, IL, USA).

### Treatment of T. brucei-infected mice with Z-Phe-Ala-CHN_2_

Female BALB/c mice (about 10 weeks old) were infected intraperitoneally (i.p.) with 2 × 10^4 ^cells of the pleomorphic *T. brucei *variant clone AnTat 1.1 [26]. On days 3 and 4 post infection (p.i.) mice were treated once daily with i.p. injections of 250 mg kg^-1 ^of Z-Phe-Ala-CHN_2 _dissolved in 70% DMSO/30% physiological NaCl solution. Infected control mice received only the vehicle. On day 5 p.i., blood smears were prepared or parasites were purified from blood by DEAE-cellulose chromatography [27].

### Staining of blood smears

Blood smears were stained with May-Grünwald's stain solution and additionally treated with 0.0001% DAPI to label the nucleus and the kinetoplast. The stained slides were examined under a microscope (Axioplan) using a 100X Plan-Neofluar objective in transmitted and fluorescence light.

### Electron microscopy

Purified trypanosomes were fixed in 2% formaldehyde/0.05% glutaraldehyde in PBS for 60 min. After embedding in 1% agarose, cells were post-fixed with 1% osmium tetraoxide/0.9% ferricyanide in PBS for 60 min followed by 1% uranyl acetate for 60 min. Cells were dehydrated in ethanol and subsequently embedded in epoxy resin. Ultrathin sections were stained with uranyl acetate and lead citrate, and examined with a Philips 201 electron microscope at 60 kV.

### Protein assay

The protein content of trypanosomes was determined using the bicinchoninic acid (BCA) method. Lysed trypanosomes (2.5 – 3.7 × 10^5 ^cells in 10 μl) were incubated with 200 μl of BCA Protein Assay reagent at 37°C. A series of dilutions of BSA (0.1 – 0.9 mg ml^-1^) was used to generate a standard curve and each dilution was set up in duplicate. After 30 min of incubation, the absorbance at 500 nm was determined using a Dynatech MR5000 ELISA reader.

## Competing interests

The authors declare that they have no competing interests.

## Authors' contributions

S.S., Y.-D.S., C.R.C. and D.S. carried out the experimental work. D.S. conceived the study and supervised its execution. D.S. and C.R.C. prepared the final draft of the manuscript. All authors have read and approved the final manuscript.
